# Serological Diagnosis and Follow-Up of Human Cystic Echinococcosis: A New Hope for the Future?

**DOI:** 10.1155/2015/428205

**Published:** 2015-10-04

**Authors:** Raúl Manzano-Román, Carlos Sánchez-Ovejero, Ana Hernández-González, Adriano Casulli, Mar Siles-Lucas

**Affiliations:** ^1^Instituto de Recursos Naturales y Agrobiología de Salamanca (IRNASA-CSIC), Cordel de Merinas 40-52, 37008 Salamanca, Spain; ^2^Instituto de Salud Carlos III, Centro Nacional de Microbiología, Majadahonda, 28220 Madrid, Spain; ^3^Istituto Superiore di Sanità, Viale Regina Elena 299, 00161 Rome, Italy

## Abstract

Cystic echinococcosis (CE) is an important helminthic zoonotic disease caused by the *Echinococcus granulosus* complex. In humans, CE is a chronic disease driven by the growth of echinococcal cysts in different organs. Prognosis of this disease depends on multiple factors, including location, number, size, and stage of the cysts, making CE a disease of complex management. CE is usually asymptomatic for years and attracts limited attention from funding organizations and health authorities. For this reason, only experts' recommendations are available but no evidence-based conclusions have been drawn for CE clinical management. One of those pitfalls refers to the lack of evidence to support the use of serological tools for the diagnosis and follow-up of CE patients. In this respect, crude antigens are used to detect specific antibodies in patients, giving rise to false positive results. The advent of molecular techniques allowing the production of recombinant proteins has provided a number of candidate antigens that could overcome the problems associated with the use of crude parasite extracts in the serological assays. In this review, we present the last advances in this field, proposing the use of serology to support cyst stage-specific diagnosis and follow-up.

## 1. Introduction

Cystic echinococcosis (CE) is a parasitic disease caused by the larval stage (metacestode) of* Echinococcus granulosus* complex which affects livestock, wildlife, and humans. CE has a worldwide geographic distribution, remaining highly endemic in many pastoral communities, including several European countries [1]. CE global prevalence is estimated at 2-3 million human cases and a burden of 1 million DALYs accounting for underreporting [2]. In humans, CE is a chronic disease characterized by the long term growth of hydatid cysts in internal organs, mainly liver and lungs, with a complex clinical management. CE results in severe and life-threatening complications, with estimated mortality rates of 2–4% per 100.000 inhabitants [3, 4]. Many CE cases are asymptomatic for years and its diagnosis is still challenging due to the absence of pathognomonic signs. For this reason CE is frequently underdiagnosed and detected only when complications arise or by chance. Additionally, the clinical management of CE (i.e., surgery, percutaneous treatment, and/or chemotherapy) has many associated risks for relapses, pointing out the importance of the follow-up of patients.

CE diagnosis and monitoring firstly rely on imaging techniques. Ultrasonography (US) standardized classification of stage-specific cystic images has been issued by the WHO Informal Working Group on Echinococcosis (WHO-IWGE) for the diagnosis and the clinical management of CE [5]. Effective serological tests for CE diagnosis would be of great help to define and support cyst status and their evolution (active: CE1, CE2, and CE3b, transitional: CE3a, or inactive: CE4 and CE5) [5, 6]. The main serological methods used for human CE diagnosis and follow-up are based on the detection of specific IgG antibodies. In this context, a number of drawbacks have been detected, including low sensitivity/specificity (Se/Sp) and a poor prognostic value for follow-up due to the long-lasting persistence of antibodies against hydatid fluid (HF) [7]. These pitfalls lead clinicians to consider serology against HF as an approach of little value, with doubtful benefit for the clinical management of CE. Alternative methods based on the detection of other antibody isotypes and IgG subisotypes against HF have been published [8]. Additionally, many authors have focused their research both on recombinant proteins and on synthetic peptides, to develop more sensitive and specific tests. Numerous recombinant proteins (Rec) and related peptides, mainly derived from the antigen B and antigen 5, have been tested for the detection and follow-up of antibodies in correlation with US findings. Unfortunately, available data were generated from small and underpowered clinical studies that have showed dissimilar Se and Sp for the same recombinant antigen [9]. Nevertheless, there are hints showing that some antigens are differentially expressed in different cyst stages, and thus antibody levels against these antigens could be associated with cyst activity and posttreatment outcome (i.e., surgery or chemotherapy) and could be applied for diagnosis and follow-up of CE patients [9, 10]. In this context, a better characterization and standardization of each antigen should be performed to clearly define its role within CE serology.

In this paper we summarize the current knowledge on the use of HF for human CE diagnosis. Additionally, results obtained from different purified fractions of parasite antigens, recombinant antigens, and synthetic peptides are also revised. A comprehensive review of the different available antigens and their performance in the diagnosis of CE was published by Carmena and colleagues [11]. In this review, we also update the findings about the available serological tools from 2006 to date.

## 2. Hydatid Fluid

HF is a complex mixture of parasite-derived proteins, mainly produced by the germinal layer of the cyst. Some of the HF components have been characterized as highly immunogenic, reaching the host environment and triggering antibody responses. The HF is the main antigenic component in the majority of commercially and in-house serological assays. This antigen mixture is used in several techniques such as the enzyme linked immunosorbent assay (ELISA), the indirect haemagglutination test (IHA), and the immunoblotting (IB). Both the ELISA and the IHA are usually the first line tests for CE patients, while the IB is used as confirmatory test. As mentioned, the use of HF for the detection of CE specific antibodies is limited by several drawbacks. First, a percentage of CE patients are serological negative against HF. Specifically, the use of HF for the detection of total IgG in ELISA test leads to variable results regarding Se and Sp. In Table 1, a number of recent studies that used IgG-ELISA tests for CE diagnosis are shown. Se reported in these studies varied from 64.8% to 100%. Reasons for false negative results depend on several factors comprising cyst location other than the liver [12, 13], early (CE1) and inactive (CE4 and CE5) cyst stages [14–16], serum collection before treatment [15, 17], single and small cysts [17], and HF antigenic source variability [18]. Additionally,* E. granulosus* complex comprises several genotypes that potentially express different antigenic sets. For instance, it has been shown that the G1 and G2 genotypes from Europe, contrary to those from China, express high quantities of antigen B2 in HF [19]. This may lead to different diagnostic performance of a specific HF. The differential expression of antigens is not only a qualitative matter, but also quantitative, depending on cyst stages. Recently, a proteomic and immunoproteomic study has shown that CE1 and CE2 cyst stages differ in the expression of their immunodominant antigens (antigen B and antigen 5). Antigen 5 is predominant and recognized by antibodies from patients with early cyst stages, while antigen B is the most scarce in CE1 cyst stage and mainly detected in patients with CE2 and CE3 cyst stages [20]. These differences might be useful in clinical practice to correctly define cyst stages and their viability by using the most indicated antigen for a stage-specific diagnosis. A second problem using HF is the percentage of false positive results detected. For instance, IgG-ELISA tests based on HF as antigen give rise to low false positive results in healthy donors (e.g., Sp from 87.5% in Indian donors to 100% in Italian donors) [15, 21]. Cross-reactivity is quite high in patients with other parasitic diseases, such as alveolar echinococcosis (AE) and cysticercosis, but also schistosomiasis and fascioliasis [22, 23]. For instance, the cross-reactivity of* E. granulosus* HF with antibodies from AE patients can reach more than 50% [17]. The HF has also been shown not to be a good antigen for patients' follow-up during the clinical management of CE. During the follow-up, ELISA-IgG test is difficult to interpret [24], and anti-HF IgG antibody reactivity may remain high many years after successful cyst removal [25].

The detection of antibodies other than IgG has shown some promising results in relation to cyst activity, relapses, and follow-up. It has been shown that both IgG2 and IgG4 could be related to cyst stages, disease evolution, and relapses [9, 26, 27]. Remarkably, it is known that the subisotype responses against CE1, CE2, and CE3 cyst stages are mainly IgG4, while IgG1, IgG2, and IgG3 responses predominate against CE4 and CE5 cysts, although this is still a question of debate [11, 22]. Antibody isotypes different than IgG can be also detected against HF in CE patients. IgE and IgM antibodies have been considered as better markers than IgG after chemotherapy and surgery [28]. Nevertheless, these isotypes are more frequently underdetected in CE patients, similar to different IgG subisotypes [29–31].

## 3. Antigens Derived from Hydatid Fluid

### 3.1. Antigen B

In an attempt to overcome the problems related to the use of HF for the detection of antibodies in CE patients, many authors have described the production and the use in serological tests of partially purified native antigens, recombinant antigens, and synthetic peptides. These are mainly represented by the two most immunogenic antigens in HF: antigen B (EgAgB) and antigen 5 (EgAg5).

EgAgB is a polymeric protein of 120–160 kDa that dissociates under reducing conditions in 8, 16, and 20–24 kDa subunits. Its biological role includes the protease inhibitor activity, neutrophil chemotaxis inhibition, triggering of nonprotective Th2 responses, induction of apoptosis of immune cells, and sequestration of xenobiotics [22]. EgAgB is codified by a multigenic family, with at least five genetic groups: EgAgB1, EgAgB2, EgAgB3, EgAgB4, and EgAgB5 [32–35]. These different subunits share from 44% to 81% of sequence identity. Each isoform has more than 90% homology with* Echinococcus granulosus* and* E. multilocularis* species, and although lower, similar antigens are also found in the genus* Taenia* [11]. These homologies can give rise to cross-reactivity with other helminthic parasites. EgAgB has been obtained as a native purified antigen, in its recombinant form. The purified native protein has a Se ranging from 60% to 85% in ELISA test and from 60% to 92% in immunoblot [11]. From 2006 up to date, several publications showed a high variability in the Se of this purified antigen, ranging from 54% to 100% (Table 2). Different cyst stages and their follow-up in US may reflect different profiles of antigens produced and released by the host immune system [20]. For instance, EgAgB recombinant antigen, in ELISA test, showed a Se of 74%, 96%, 90%, and 56% in patients with CE1, CE2, CE3, and CE4/CE5 cyst stages, respectively [36]. Additional reasons for false negative results have been pointed out by several authors, including cyst localization other than liver and small size of cysts (see Table 2). These results are similar to those found for false negative reactions against HF. The heterogeneity and thus the limited value of native antigens are also found when the purified EgAgB is used for the serodiagnosis of CE patients. In this respect, antigens purified from different sources of HF result in a variable Se in the same patients' cohort (from 82.1% to 96.9%; [37]; Table 2).

In its recombinant form (Rec), mainly two isoforms have been assayed: RecEgAgB1 and RecEgAgB2. RecEgAgB1 gives rise to variable sensitivities, ranging from 55% to 84% [11] and from 71% to 94.6% in subsequent studies [38] (Table 2). The lowest Se (71%; [38]) was attributed to false negative results due to patients with* Echinococcus* genotypes other than G1. Nevertheless, additional factors such as the presence of CE4 and CE5 cyst stages and serum sampling collection with respect to treatment are probably contributing to false negative results against this recombinant antigen [15]. Some of these factors have been also pointed out for RecEgAgB2, for which false negatives have been related to single cysts and to sera collected before treatment with benzimidazoles [17]. RecEgAgB2 has given rise to very variable sensitivities as well ([11]; Table 2). A modified version of the RecEgAgB2, consisting of twofold tandem repeat of the original recombinant protein, showed a Se similar to the single antigen unit [17]. Additional isoforms of EgAgB have been tested on few occasions (Table 2). RecEgAgB3 and B5 did not improve the Se of other antigenic isoforms [19], but RecEgAgB4 has shown promising results (Se from 75.8% to 91.7%) [19, 39]. Remarkably, EgAgB3 and B5 were not found after a proteomic study on the composition of the HF in CE1 and CE2 cyst stages from sheep [40].

As mentioned, several synthetic peptides derived from EgAgB1 have been also tested in ELISA for the detection of specific antibodies. From those tests, the most promising results have been obtained with the p176 peptide ([11]; Table 2). Nevertheless, the diagnostic performance of synthetic peptides has been worse than that of the whole original molecule, either in its purified native or in recombinant forms. In addition, pooling three peptides has not resulted in the improvement of the test Se [41]. In any case, the use of bioinformatics for peptides selection and their screening in high-throughput platforms have been stated as a proof of principle and this approach could be used in the future for the identification of relevant diagnostic epitopes. As mentioned, a dominant IgG4 response is usually associated with active and transitional cysts, whereas inactive cysts mainly trigger IgG1, IgG2, and IgG3 responses. This has important implications in the interpretation of the usefulness of the detection of different IgG isotypes, since the level of reactivity of each subisotype could be driven by the cyst stages found in patients. This is the case for IgG4, since the Se obtained when detecting this subisotype is lower than that detected when total specific IgG levels are evaluated [42]. Whether this lower Se is associated with inactive cysts, it should be further evaluated to ascertain the usefulness of IgG4 instead of total IgG, both for the diagnosis and for the follow-up of CE patients. The Sp of EgAgB in its different isoforms is also variable, although the main cross-reactivity has been reported with patients affected by AE and cysticercosis [11]. Additionally, antibodies from patients with other parasitic diseases including onchocerciasis, schistosomiasis, and toxocariasis have given rise to false positive reactions when tested against EgAgB [11]. It is worth mentioning the efforts of several authors to test new affordable and easy to use serological techniques instead of ELISA or the IB tests, making those immunoassays stand out based on particles such as DIGFA (dot immunogold filtration assay [43, 44]; Table 2).

In the light of the results obtained by several authors using EgAgB, this antigenic family looks promising for the serodiagnosis of CE patients and potentially useful in the follow-up. Anyway, testing of this antigen family obtained in a standardized format, with a well-defined and wide panel of CE samples, including the clinical information influencing the interpretation of the test (the parasite genotype, location, number, size, and type of cysts, and presence of complications and coinfections), should be further performed.

### 3.2. Antigen 5

Antigen 5 (EgAg5) is also an abundant highly immunogenic component of HF. EgAg5 is a thermolabile protein of around 400 kDa, composed of two subunits of 57 and 67 kDa. Studies on the N-terminal sequence of the 38 kDa subunit have shown that several isoforms exist similar to EgAgB, and thus EgAg5 could be also coded by a multigenic family [45]. The biological function of EgAg5 is largely unknown, and although the 38 kDa subunit shows homologies with trypsin peptidases, the enzymatic activity is lacking [46]. Additionally, the smallest subunit of 22 kDa contains proteoglycans of the heparan sulfate group and calcium-binding motifs, suggesting that this subunit could interact with the extracellular matrix and the cell surface [47].

Molecules very similar to EgAg5 (more than 90% identity) are also expressed by* E. multilocularis* and the genus* Taenia*; thus cross-reactivity of this antigen with antibodies from patients affected by AE and cysticercosis is expected. As we explain in the following paragraphs, the diagnostic use of EgAg5 has shown mainly drawbacks regarding Se and Sp. The purified native EgAg5 has been obtained using different methodologies, including size exclusion chromatography and immunoaffinity. Lately, an easy and efficient method for the enrichment of this antigen from HF has been described [48]. Variability of its performance in serodiagnosis could be also attributed to differences in the clinical status of the patients. For instance, EgAg5 seems to play a role in the induction of specific antibodies mainly when CE1 and CE5 cyst stages are present [40]. This variable Se has been reported by several authors, ranging from 50% to 87.5% [11, 49]. As recombinant proteins, EgAg5 has been tested in its full format (RecEgAg5) and in its 38 kDa version (RecEgAg53.38) by Auer and colleagues [50]. Although the 38 kDa subunit has been described as the most antigenic component detected by IB, the Se of the corresponding recombinant antigen was very low (21%) [50, 51]. Similarly, low and variable sensitivities (from 16% to 85%) have been detected using the p89-122 EgAg5 peptide [11]. Due to the low Se of EgAg5 and derivatives, this antigen has been less frequently tested than EgAgB. Nevertheless, reasons for this low Se could be due to cyst stage; thus the usefulness of this antigen for the detection of early active and late inactive cyst lesions should be further evaluated.

### 3.3. Other Antigens

The most frequently used crude extract of* E. granulosus* for the serodiagnosis of CE has been the somatic extract of protoscoleces (EgPpsSom). This extract has a Se ranging from 69.4% to 96.9% [11, 16, 43, 44, 52–54]. When compared with HF, the EgPpsSom performs worse. Less frequently, other somatic extracts have been applied in the serodiagnosis of CE patients such as those derived from the cyst wall (96.7% Se; [52]), the tegument of protoscoleces (81.3% Se; [16]), and the adult worm (82% Se; [55]). Cross-reactivity of those extracts is similar to that detected in HF. Drawbacks associated with the heterogeneity of somatic extracts could be attributed to these antigenic preparations. In addition, reasons for the presence of false negative results are similar to those found when the HF is used as antigenic source (cyst location other than liver, small size cysts, and CE1, CE4, and CE5 cyst stages).

Additionally, several recombinant antigens different from EgAgB and EgAg5 have been obtained by several authors and tested for the diagnosis of CE. These include, among others, the malate dehydrogenase (RecEgMDH), the calcium binding protein (RecEgCaBP), the actin filament fragmenting protein (RecEgAFFP), the RecEgEpC1, the thioredoxin peroxidase (RecEGTPx), and the RecEg19 [11, 56, 57]. RecEGTPx and RecEg19 showed low Se (<45%; [56–58]). The rest of the above-mentioned antigens showed variable Se (even for the same recombinant) ranging from 45% (MDH) to 90% (MDH, EpC1) and variable Sp (even for the same antigen) ranging from 83% to 95% [11]. For this reason these antigens could be considered as good alternatives for serological tests, although further characterization is needed to evaluate their diagnostic potential.

## 4. Antigens for Clinical Management

The clinical management of CE should be based on recommendations done by experts and driven by cystic stages identification [54]. In this context, the WHO-IWGE has proposed a cyst classification based on active, transitional, and inactive cysts.

Some antigens could be mainly expressed by defined cyst stages, showing that serology could be potentially useful for the definition of cyst activity and thus for the clinical management of CE patients. As mentioned, the HF has been also evaluated for the follow-up of patients treated by surgery and/or chemotherapy, but its usefulness is hampered by the long persistence of antibodies in patients with nonactive cysts [59]. A better correlation of specific antibodies against defined active cyst stages has been reported in patients after surgical treatment, including EgAgB [59, 60], EgAgP29 [60], and the heat-shock protein 20 [61]. Several authors have also found a correlation between a low level of specific antibodies against RecEgAgB2, RecEgAgHSP20, and RecEgAgB1 and the presence of inactive cysts [36, 59, 61]. Nevertheless, some of those antigens are not recognized by a percentage of CE patients [60]. Additionally, it has been shown that the banding pattern in IB from CE patients changes depending on the cyst stage [62].

Similarly, other authors have detected the loss of defined bands in IB after cure. The identification and characterization of the above-mentioned antigens could support the clinical decision making actually based on imaging techniques. Similarly, the shift of defined subisotypes of IgG antibodies during cyst evolution could be used for the follow-up of CE patients. As mentioned, IgG4 levels could be correlated with cyst activity, and declining of the levels of isotypes other than IgG seems to be useful to define the success of treatment [26, 27]. Nevertheless, detectable levels of specific IgG4 and especially of IgE and IgM are only found in a relative small percentage of CE patients.

## 5. Conclusions: A New Hope for the Future?

Diagnosis and follow-up of CE patients are mainly based on imaging techniques. These can be used for the identification of cystic stages, leading to a stage-specific approach in CE clinical management. Serological tools supporting imaging techniques would be desirable. However the available tests are based on antibodies against crude antigens and thus marred by poor Sp and Se, with low or no usefulness for the follow-up of patients during the treatment. Specific recombinant antigens have good potential as diagnostic and follow-up tools for CE, but progress in this field is hampered by lack of standardization. Thus, a challenge still exists to develop a reliable world standard based on serology for the diagnosis and monitoring of CE patients. In this respect, a multicentre study with a wide panel of sera from CE patients, including relevant clinical data to properly define the usefulness of the available recombinant antigens, should be performed. Along this path, a project focusing on CE has been recently funded by the European commission under the Seventh Framework Programme (HERACLES; http://www.heracles-fp7.eu/). In the framework of HERACLES, six recombinant antigens (RecEgAgB1, RecEgAgB2, RecEgAg5, RecEgAgMDH, RecEgAgCaBP, and RecEgAgAFFP) will be tested for the serodiagnosis and follow-up of CE on a wide panel of samples obtained from extended ultrasound surveys in Eastern Europe. These recombinant antigens have been already produced in a standardized way and preliminary tested in ELISA for the detection of specific IgG in sera. Additionally, two databases for the collection of clinical data from retrospective/prospective patients providing serum samples have been developed (the database behind the European Register of Cystic Echinococcosis ERCE, http://www.heracles-fp7.eu/erce.html, and CYSTRACK database, http://cystrack.irnasa.csic.es). Hosting of samples and clinical data has been organized in a dedicated biobank (EchinoBiobank). This strategy will hopefully pave the way to improve the diagnosis and follow-up of CE patients, providing evidence-based data on the usefulness of serology in CE clinical management.

## Figures and Tables

**Table 1:** Performance of the hydatid cyst fluid in ELISA test for the detection of total IgG in CE patients (articles published from 2006).

Number of CE patients	Confirmatory test	Sensitivity (%)	Negative serology more frequent when	Reference
23	Histopathology	100	Not specified	[40]
5	Imaging techniques plus serology	80	Not specified	
13	Serology	100	Not specified	

41	Surgery	95.1	Not specified	[21]

6	Imaging techniques	66.7	Cyst location other than liver	[12]

144	Imaging techniques	92.4	CE1, CE4, and CE5 cyst stages	[14]

123	Imaging techniques	64.8^*∗*^	CE4 and CE5 cyst stages, no pretreatment	[15]

59	Surgery	95.1	Not specified	[20]

10	Surgery	100	Not specified	[52]

54	Surgery	81.5	Single cyst, no pretreatment	[17]
186	Surgery	83.3		

32	Imaging techniques	93.8	CE4 and CE5 cyst stages	[16]

155	Surgery	90.3^*∗*^	Not specified	[29]

40	Surgery	92.5	Not specified	[30]

47	Surgery	95.7^1^	HF collected from cysts of different hosts or of different anatomical locations from the same host	[18]
		93.6^2^		
		91.4^3^		
		97.8^4^		
		93.6^5^		
		78.5^6^		
		72.2^7^		

63	Surgery	90.5	Not specified	[48]
		82.4^*∗*^		

68	Imaging techniques	92.6	Lung cysts	[13]

^*∗*^Commercial test; HF of cysts from ^1^sheep liver, ^2^sheep lungs, ^3^goat liver, ^4^human liver, ^5^camel lungs, ^6^cow lungs, and ^7^cocktail.

**Table 2:** Use of antigen B and derivatives for the diagnosis of CE patients (articles published from 2006).

Antigen	Number of CE patients	Confirmatory test	Technique, antibody	Sensitivity (%)	Negative serology more frequent when	Reference
Purified	21	Surgery	Immunochromatography, IgG	100	Not specified	[42]
			Immunochromatography, IgG4	95		
	23	Surgery	ELISA, IgG	100	Not specified	[46]
	5	Imaging techniques and serology^*∗*^		80		
	13	Serology^*∗*^		0		
	32	Imaging techniques	ELISA, IgG	93.8	CE4 and CE5 cyst stages	[16]
	40	Surgery	ELISA, IgG	87.5	Not specified	[30]
			ELISA, IgG4	80		
	108	Surgery	DIGFA, IgG	89.8	Cyst location other than liver; CE1, CE4, and CE5 cyst stages; small cysts	[43]
	113	Imaging techniques	DIGFA, IgG	92.9	Not specified	[44]
	35	Imaging techniques	ELISA, IgG	54	CE1, CE4, and CE5 cyst stages	[63]
	56	Surgery	ELISA, all (Protein G)	96.9^1^	Not specified	[37]
				82.1^2^		

Recombinant B1	31	Surgery	ELISA, all (Protein G)	71	Parasite genotype other than G1	[38]
	124	Surgery^$^	ELISA, IgG	83	Not specified	[19]
	246	Imaging techniques	ELISA, all (Protein G)	77.6	Not specified	[64]
	113	Imaging techniques	DIGFA, IgG	77.9	Not specified	[44]
	56	Surgery	ELISA, all (Protein G)	94.6	Not specified	[37]
	123	Imaging techniques	ELISA, IgG	73.9	CE4 and CE5 cyst stages, no pretreatment	[15]

Recombinant B2	54^3^	Surgery	ELISA, IgG	77.8	Single cyst, no pretreatment	[17]
	186^4^			79		
	124	Surgery^$^	ELISA, IgG	62.9	Not specified	[19]

Recombinant 2B2	54^3^	Surgery	ELISA, IgG	92.6	Single cyst, no pretreatment	[17]
	186^4^			87.6		

Recombinant B3	124	Surgery^$^	ELISA, IgG	29	Not specified	[19]

Recombinant B4	124	Surgery^$^	ELISA, IgG	75.8	Not specified	[19]
	36	Surgery	ELISA, IgG	91.7	Not specified	[39]

Recombinant B5	124	Surgery^$^	ELISA, IgG	41.3	Not specified	[19]

P176 peptide	61	Surgery	ELISA, IgG	78.7	Lung cysts, no complications, single cyst, and small cysts	[48]
	63	Surgery	ELISA, IgG	23.8	Not specified	[50]

Long D8-9 peptide	35	Imaging techniques	ELISA IgG	74.3	Not specified	[41]

^*∗*^Positive serology against HF in ELISA IgG, ^1^EgAgB purified from a Chinese sheep isolate, ^2^EgAgB purified from a Iranian sheep isolate, ^$^patients selected by their positivity in ELISA IgG against HF, ^3^Spanish patients, and ^4^Peruvian patients.
